# Can atopic eczema and psoriasis coexist? A systematic review and meta‐analysis

**DOI:** 10.1002/ski2.29

**Published:** 2021-05-05

**Authors:** A. Cunliffe, S. Gran, U. Ali, D. Grindlay, S. J. Lax, H. C. Williams, E. Burden‐Teh

**Affiliations:** ^1^ Nottingham University Hospitals NHS Trust Nottingham UK; ^2^ Centre of Evidence Based Dermatology School of Medicine University of Nottingham Nottingham UK

## Abstract

**Importance:**

Previous studies report both coexistence and mutual exclusivity of atopic eczema (AE) and psoriasis, but these have not been appraised systematically. Knowledge of such disease association throws light on disease mechanisms and may influence therapeutic choices.

**Objective:**

To summarise evidence for AE and psoriasis occurring in the same person at the same point in time. Planned primary outcome was the incidence, prevalence or risk of psoriasis or eczema.

**Methods:**

Ovid MEDLINE and Ovid Embase were searched from inception to 1st February 2020. The search strategy was built around the key terms ‘atopic eczema’, ‘psoriasis’ and ‘co‐existence’. Observational studies (cohort, case‐control, cross‐sectional and case‐series) with a minimum of 10 consecutive patients were included. There were no restrictions on participants, geography or language. Studies were selected, data extracted and critically appraised by two independent reviewers. Data were extracted on the method of diagnosis: health professional (dermatologist, criteria, other), self‐reported, not specified. Study quality was assessed using validated Joanna Brigg's Institute critical appraisal tools. A random‐effects model was used to combine studies. The effect of study quality on the pooled estimate was investigated using stratification. Heterogeneity was explored by subgroup analysis.

**Results:**

This review included 31 studies and 20 523 individuals with psoriasis and 1 405 911 with AE. Eight studies reported the prevalence of AE in those with psoriasis and values ranged from 0.17% to 20%: the pooled prevalence was 2% (95% confidence interval [CI]: 1, 3). Seven studies reported the prevalence of psoriasis in those with AE and values ranged from 0.3% to 12.6%; the pooled prevalence was 2% (95% CI: 1, 3). Ten studies were assessed as low risk of bias. Geographical area, method of diagnosis, setting and whether the assessment of diagnosis was blinded, partly contributed to the heterogeneity.

**Conclusions:**

This review provides some evidence for the coexistence of AE and psoriasis. Clinicians should be aware of coexistence at diagnosis, when selecting therapies and when reviewing poor response to treatment.

## Graphical abstract

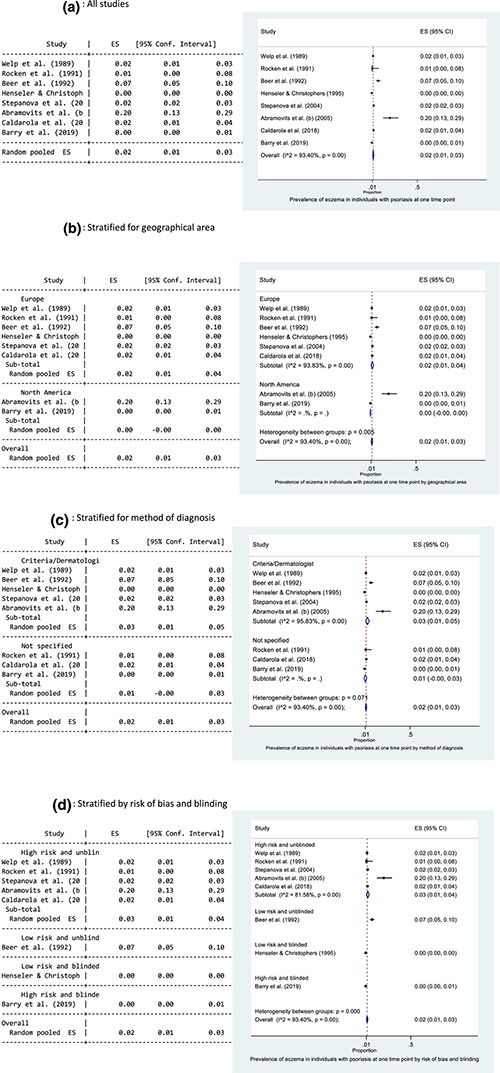

A systematic review and meta‐analysis of observational studies that reported incidence, prevalence or risk data on the co‐existence of atopic eczema (AE) and psoriasis in the same individual. Thirty‐one studies were included and the pooled prevalence of AE occurring in individuals with psoriasis and vice versa was 2%. This review provides some evidence for the co‐existence of AE and psoriasis.

1

**What is already known about this topic?**

- Atopic eczema and psoriasis are two of the most common skin diseases and are managed in primary, secondary and tertiary care.
- Observational studies have supported both coexistence and mutual exclusivity of atopic eczema and psoriasis.
- Studies also conflict on the coexistence of other Th1 and Th2 diseases.

**What does this study add?**

- There is some evidence that atopic eczema and psoriasis may present in the same individual, both simultaneously and consecutively.
- Coexistence of disease may occur at a level equal or lower than expected.
- Clinicians should be aware of coexistence at diagnosis, when selecting therapies and when reviewing poor response to treatment.

## 2 INTRODUCTION

Atopic eczema (also known as atopic dermatitis or just eczema) and psoriasis are both common chronic, inflammatory diseases affecting the skin, with large variations in their incidence and prevalence between countries.^1^, ^2^, ^3^ The individual, societal and healthcare costs of both diseases are significant. Eczema and psoriasis can affect people of any age, localise to high impact sites (e.g., hands), involve large areas of the body, effect psychosocial well‐being and have a negative impact on quality of life especially with coexistent comorbidities.^4^ ^,^ ^5^ Management of both diseases is therefore part of routine practice and the focus of highly specialised clinics.

Eczema and psoriasis are primarily diagnosed by a healthcare professional after a clinical examination. Diagnostic criteria for eczema, but not psoriasis, have been validated and are widely used in clinical research.^6^ ^,^ ^7^ It has been argued that eczema and psoriasis cannot coexist in the same person because this requires the activation of opposing inflammatory pathways (Th2 vs. Th1).^8^ ^,^ ^9^ To date, observational studies have supported both coexistence and mutual exclusivity of eczema and psoriasis, and also reported conflicting relationships between other Th2 and Th1 diseases.^10^, ^11^, ^12^

This systematic review aims to reconcile these divergent results through collectively presenting the spectrum of evidence and providing pooled values with greater precision than individual smaller studies. In the review we will explore the effect of important differences between studies (population, study design, study quality) on the results. We intend that new summative data on the coexistence of eczema and psoriasis will give important clinical or population level insight into the immunopathogenesis of both diseases. These data may inform the development and choice of targeted biologic treatments and may challenge how we clinically categorise disease.

The primary objective of this systematic review and meta‐analysis is to investigate the incidence, prevalence or risk of eczema and psoriasis occurring in the same individual at the same point in time.

## 3 METHODS

### 3.1 Protocol

The review protocol is available on PROSPERO https://www.crd.york.ac.uk/prospero/display_record.php?RecordID=118343.

### 3.2 Objectives of the review

The primary objective for the review was to investigate the prevalence, incidence or risk (relative risk or odds ratio [OR]) of eczema and psoriasis occurring in the same individual at the same time point. The secondary objective was to investigate the same at any time point. A tertiary objective was to summarise the immunologic and genetic data on coexistence.

### 3.3 Search strategy and reference searching

OVID MEDLINE and OVID Embase were searched from inception on 1st February 2020. A search strategy was built with an information specialist (D.G.) around the key terms ‘atopic eczema’, ‘psoriasis’ and ‘co‐existence’ using medical subject headings and free text words (Supporting Information 1). Reference lists of included studies were hand‐searched. Studies with a primary objective matching the review question were also included in prospective citation searching using Google Scholar. Authors of included studies were emailed to help identify any published or unpublished data on the review question.

### 3.4 Inclusion and exclusion criteria

Observational studies (cohort, case‐control, cross‐sectional and case‐series of at least 10 consecutive patients to give sufficient data) were included. Studies were required to provide incidence, prevalence or risk data on the coexistence of psoriasis and eczema. Cohort studies were included to provide data on coexistence in the same time period, consecutive onset and incidence estimates.

There were no restrictions on the age, gender, ethnicity, setting or geographical location of participants. Studies of all languages for which a translator was available were included in the review. The review investigated two separate populations: (i) the diagnosis of eczema (outcome) in a psoriasis population (exposure); (ii) the diagnosis of psoriasis (outcome) in an eczema population (exposure). The diseases of interest, eczema and psoriasis, were defined and recorded as a diagnosis made by a health professional (clinical diagnosis), self‐reported or the method of diagnosis was unspecified. The category of diagnosis made by a health professional was further divided into whether it was made by a dermatologist or nondermatologist, and if diagnostic criteria were used. The definition of psoriasis included all subtypes of psoriasis. The term atopic eczema has been used interchangeably with eczema/dermatitis and studies were excluded if they specifically focused on nonatopic eczema (e.g., irritant, allergic, venous, nummular). Conference abstracts were included if they contained sufficient information on the primary objective.

### 3.5 Study screening and eligibility assessment

Titles and abstracts were screened independently by two authors (A.C., U.A. or E.B.T.) using Rayyan QCRI (https://rayyan.qcri.org/). Full text eligibility was independently assessed by two authors (A.C., U.A. or EBT) and any differences were discussed with a third author (E.B.T. or S.G).

### 3.6 Data extraction

Two authors (A.C., U.A., E.B.T. or S.J.L.) independently completed the data extraction from included studies. A data extraction pro‐forma was piloted on five studies (Supporting Information 2). Timing of outcome was categorised as at the same time point (simultaneous disease), any time point (lifetime or study duration coexistence) or timing unclear. Where prevalence data were missing, study authors were contacted for further details.

### 3.7 Quality assessment

Included studies were critically appraised by two independent authors (A.C., U.A., E.B.T. or S.J.L.) using validated Joanna Briggs Institute critical appraisal quality assessment tools for each study type.^13^ The number of appraisal questions for each study type differed and therefore a percentage of criteria indicating low risk of bias was calculated; ≥70% was required for studies to be classified as low risk of bias. An additional quality assessment question was added by the review team post‐hoc to assess whether the outcome diagnosis of eczema and/or psoriasis was made blinded to the review question. We felt this was an important potential source of information bias, because knowledge of the research question may have influenced whether a diagnosis of coexistent eczema and psoriasis was made or not.

### 3.8 Data analysis

All extracted data were included in a narrative synthesis. A meta‐analysis for the primary and secondary objectives was planned if there were sufficient clinically similar studies providing frequency/association data. Meta‐analysis was only possible for prevalence data because there were insufficient data on incidence and risk; pooled data have been presented as forest plots with 95% confidence intervals (CIs). A random effects model (DerSimonian and Laird) was used and heterogeneity assessed statistically using standard Chi‐squared (*I* ^2^). Heterogeneity was explored by planned subgroup analysis (setting, geographical area, method of diagnosis, age of participant, risk of bias). Stata v14.0 was used for the meta‐analysis. Assessment of publication bias using Egger's test was planned, but not possible because there were less than 10 studies in each meta‐analysis.

## 4 RESULTS

**FIGURE 1:**
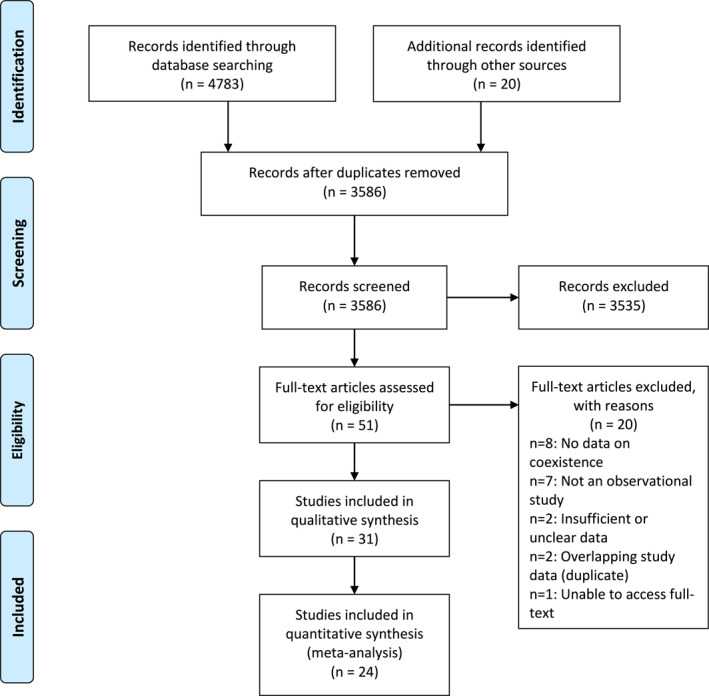
PRISMA flow diagram of included studies

The electronic database search identified 4783 citations. An additional 20 studies were identified through retrospective and prospective citation searching. No studies were identified by direct contact with study authors (three authors responded). After deduplication 3586 studies remained. Fifty‐one full text articles were assessed for eligibility and 31 were included in the review.^10^, ^11^, ^12^ ^,^ ^14^, ^15^, ^16^, ^17^, ^18^, ^19^, ^20^, ^21^, ^22^, ^23^, ^24^, ^25^, ^26^, ^27^, ^28^, ^29^, ^30^, ^31^, ^32^, ^33^, ^34^, ^35^, ^36^, ^37^, ^38^, ^39^, ^40^, ^41^ This included four conference abstracts.^19^ ^,^ ^22^ ^,^ ^24^ ^,^ ^33^ The PRISMA flow diagram is presented in Figure 1 and excluded studies in Supporting Information 3.

The characteristics of each included study are presented in Tables 1 and 2. In total, there were 25 cross‐sectional studies, three cohort and three case series. All studies were conducted between 1989 and 2020. Nineteen were conducted in Europe, four in the United States of America, four in Asia, three in the Middle East and one in Africa. Twenty‐one were hospital‐based studies, nine were population‐based studies and one was conducted in schools.

**TABLE 1:** Study characteristics of included studies

Study, year, country	Study type and setting	Aim	Diagnosis	Sample size (*n* = psoriasis population)	Timing of outcome	Population	Male and female (%)	Prevalence of eczema (%)	Other key findings	Risk of bias	Blinded assessment of outcome diagnosis
Al‐ Fouzan et al.^14^ 1994, Kuwait	Cross‐sectional hospital‐based Dermatology department	To determine the prevalence, and clinical and epidemiologic features of childhood psoriasis	Psoriasis: not specified, histology if diagnosis uncertain Eczema: not specified	190	Unclear	Children (0–12 years) Plaque psoriasis (84%)	Male: 40% Female: 60%	1% (2/190)		High risk of bias	No
Abramovits et al.^15^ 2005, USA	Cross‐sectional hospital‐based Dermatology department	To determine how frequently psoriasis patients present with features of both psoriasis and eczema.	Psoriasis: dermatologist Eczema: dermatologist (criteria for diagnosis specified)	100	One time point Any time point	Adults and children	Male: 53% Female: 47%	One time point 20% (20/100)—defined as PsEma Any time point 42% (42/100)		High risk of bias	No
Augustin et al.^17^ 2015, Germany	Cross‐ sectional population‐based German statutory health insurance database	To compare the comorbidity and prevalence data in psoriasis and eczema	Psoriasis and eczema: ICD10 codes (healthcare professional not specified)	1313	Unclear	Children (<18 years)	Male and female: % unclear	24.52% (322/1313)	Prevalence ratio of eczema in psoriasis 2.83 (95% CI 2.50–3.21)	High risk of bias	Yes
Barry et al.^18^ ^,^ ^a^ 2019, USA	Cross‐sectional hospital‐based Dermatology department	To characterise the population of patients diagnosed with both AD and psoriasis	Psoriasis and eczema: health professional (not specified) and histology (33%)	1392	One time point Any time point	Adults and children	Male and female: % unclear	At one time point 0.2% (3/1392) At any time point 2%	Concomitant 1.3% (30/2303)	High risk of bias	Yes
Beer et al.^12^ ^,^ ^a^ 1992, UK	Cross‐sectional hospital‐based Dermatology department	A prospective study to record the concurrent or consecutive coincidence of psoriasis and atopic dermatitis, and any shared clinical features	Psoriasis and eczema: dermatologist (criteria for diagnosis specified)	428	One time point Any time point	Adults and children	Male: 83% Females: 17%	At one time point 7.2% (34/473) At any time point 9.5% (45/473)		Low risk of bias	No
Ben Rejeb et al.^19^ 2019, Tunisia	Case‐series hospital‐based Dermatology department Conference abstract	To describe the paediatric psoriasis population in their department	Psoriasis and eczema: healthcare professional (not specified)	306	Unclear	Children (0–15 years) Plaque psoriasis (76%)	Male: 44% Female: 56%	30%		High risk of bias	Yes
Caldarola et al.^20^ 2019, Italy	Cross‐sectional hospital‐based Dermatology department	To measure the prevalence of cutaneous comorbidities in adult patients with plaque psoriasis and assess their impact on quality of life	Psoriasis and eczema: health professional (not specified)	560	One time point	Adults Mostly moderate to severe psoriasis	Male: 56% Female: 44%	2.1% (12/560)		High risk of bias	No
Christensen et al.^21^ 2006, USA	Cross‐sectional hospital‐based Dermatology department	To describe the associated phenotypic features of patients with three types of plaque thickness	Psoriasis: dermatologist Eczema: self‐reported	500	Any time point	Adults and children Caucasian (93%)	Male: 50% Female: 50%	11.6% (58/500)		High risk of bias	Yes
Ejaz et al.^23^ 2013, Pakistan	Cross‐sectional hospital‐based Dermatology department	To observe the clinical features, laboratory profile, associations, and comorbidities of psoriasis	Psoriasis: dermatologist Eczema: Self‐reported	100	Unclear	Adults and children Plaque psoriasis (88%) Pakistani ethnicity	Male: 71% Female: 29%	3% (3/100)		High risk of bias	No
Galili et al.^23^ 2017 Israel	Cross‐sectional population‐based Data source not specified Conference abstract	To investigate the association between psoriasis and atopic diseases among adolescents	Psoriasis: dermatologist Eczema: not specified	3122	Unclear	Adolescents (16–18 years)	Males and females: % unclear	1.57% (49/3122)	Prevalence of eczema in controls 0.74% Adjusted OR of eczema in psoriasis 1.75 (95% CI: 1.29, 2.37), adjusted for age, sex, country of origin, socioeconomic status and body mass index	High risk of bias	Yes
Garofalo et al.^25^ 1989, Italy	Cross‐sectional hospital‐ based Dermatology department	To specify the incidence of atopic diathesis in children with psoriasis	Psoriasis: not specified Eczema: Hanifin and Rajka diagnostic criteria	589	Any time point	Children (<12 years) Plaque psoriasis (67%)	Male: 45% Female: 55%	4% (24/589)		High risk of bias	No
Henseler & Christopher,^10^ ^,^ ^a^ 1995, Germany	Cross‐sectional hospital‐based Dermatology department	To determine the frequency of concurrent diseases	Psoriasis and eczema: dermatologist	2941	One time point	Age not specified Hospitalised	Male: 51% Female: 49%	0.17% (5/2941)	Observed/expected ratio of eczema in psoriasis 0.04	Low risk of bias	Yes
Hosseini et al.^26^ 2019, Iran	Cross‐sectional hospital‐based Dermatology department	To investigate the association between psoriasis and atopy	Psoriasis: dermatologist and histology Eczema: ISAAC questionnaire (based on UKWP diagnostic criteria)	52	Any time point	Adults Plaque psoriasis (69%) Excluded patients on immunosuppressive drugs	Male: 36.5% Female: 63.5%	26.9%	Prevalence of eczema in controls 26%	Low risk of bias	No
Kim et al.^27^ ^,^ ^a^ 2010, South Korea	Cross‐sectional population‐based Health centre attendance for an annual health check	To determine the prevalence of atopic dermatitis in Korean adults and also the coincidence of psoriasis	Psoriasis: dermatologist and self‐ reported Eczema: dermatologist and self‐ reported, both based on Hanifin and Rajka criteria	104	Any time point	Adults Korean ethnicity	Male and female: % unclear	9.6% (10/104)	Prevalence coexistent disease in the whole population 0.5% (10/2032)	Low risk of bias	No
Lambert & Dalac,^29^ ^,^ ^a^ 1992, France & Belgium	Cross‐sectional hospital‐based Dermatology department	To look at the association between psoriasis and eczema	Psoriasis: dermatologist Eczema: dermatologist using diagnostic criteria	3837	Unclear	Age not specified	Male and female: % unclear	2.5% (95/3837)		High risk of bias	No
Landgren et al.^11^ 2005, Sweden	Cross‐sectional population‐ based Swedish military service conscription register	To determine the prevalence of psoriasis in young Swedish men over a period of three decades and the association between psoriasis and allergic disorders	Psoriasis and eczema: healthcare professional (medical doctor) and self‐reported	Number with psoriasis not specified (Total study population 1226 193)	Unclear	Adults (17–20 year olds) Military conscripts	Male: 100%		Risk ratio of eczema in psoriasis 0.40 (95% CI: 0.30, 0.55)	Low risk of bias	Yes
Naldi et al.^30^ ^,^ ^a^ 2009, Italy	Cross‐sectional community‐based Secondary schools	To estimate the lifetime risk of atopic dermatitis in children and to quantify the association between selected diseases	Psoriasis and eczema: health professional (from the medical record)	68	Any time	Children (12–17 years) Attending school Caucasian	Male and female: % unclear	23.5% (16/68)	Rate ratio 5.5 of eczema in psoriasis (95% CI: 3.0, 10.1)	Low risk of bias	Yes
Pigatto,^31^ 2000, Italy	Cross‐sectional hospital‐based Dermatology department	To investigate the incidence of allergic sensitisation in patients with psoriasis	Psoriasis: health professional (not specified) Eczema: not specified	140	Unclear	Adults Stable plaque psoriasis (64%)	Male: 14% Female: 86%	0%		High risk of bias	No
Rocken et al.^32^ 1991, Germany	Cross‐sectional hospital‐based Dermatology department	To find the prevalence of atopy in inpatients with psoriasis	Psoriasis and eczema: not specified	68	One time point	Age not specified Hospitalised	Male: 65% Female: 35%	1.5% (1/68)	No other evidence of atopy (total IgE <100 kU/L and RAST test to inhaled allergens normal)	High risk of bias	No
Salavastru,^33^ 2016 Romania	Case‐series Hospital‐based Dermatology department Conference abstract	To characterise the clinical features of paediatric psoriasis	Psoriasis and eczema: not specified	125	Unclear	Children (12–18 years) Plaque psoriasis	Male and female: % unclear	18.4% (23/125)		High risk of bias	Yes
Stepanova et al.^35^ ^,^ ^a^ 2004, Germany	Cross‐sectional hospital‐based Dermatology department	To report the occurrence of ‘mixed forms’ of eczema and psoriasis, and verify the immunological relationship, total IgE value and distribution of the HLA characteristics	Psoriasis: diagnostic criteria (not specified who made the diagnosis)* Eczema: Hanifin and Rajka (not specified who made the diagnosis)* ‘Mixed form’ defined as ‘atopic dermatitis criteria’ as well as the ‘psoriasis criteria’ *method of diagnosis only specified for exposure	1216	One time point	Adults Hospitalised	Male and female: % unclear	2.4% (29/1216)	42.5% (34/80) of ‘mixed form’ have elevated total IgE >120 kU/L ‘Mixed form’ in females demonstrated homozygosity of the region between HLA‐B and DRB1: DRB4 (DR53)	High risk of bias	No
Toscano et al.^36^ 2014, Italy	Cross‐sectional hospital‐based Paediatric department	To examine the prevalence and pattern of presentation of paediatric psoriasis	Psoriasis and eczema: not specified	66	Unclear	Children (<14 years) Plaque psoriasis (67%)	Male: 52% Female: 48%	18.2% (12/66)		High risk of bias	Yes
Unal et al.^37^ 2017, Turkey	Cross‐sectional	To determine the association of atopy with patient history	Psoriasis: health professional (not specified) Eczema: health professional (not specified) and self‐reported	94	Any time point	Adults	Male and female: % unclear	0%		High risk of bias	No
Welp et al.^38^ 1989, Germany	Cross‐sectional hospital‐based Dermatology department	To determine the coincidence of psoriasis and eczema	Psoriasis: not specified+/‐ histology Eczema—Hanifin and Rajka (not specified who made the diagnosis), +/− histology	1065	One time point	Adults	Unclear	1.7% (18/1065)	89% (16/18) of the patients showed a total IgE level of over 120 IU/ml Significant increase in HLA‐Cw6 in 50% (9/18) patients, while HLA‐B7 and HLA‐B17 were only found in 11% (2/18) patients.	High risk of bias	No
Wu et al.^41^ 2010, China	Case‐series Hospital‐based Dermatology department	To characterise the population of paediatric psoriasis at their centre	Psoriasis: criteria used but no details specified Eczema: not specified	137	Unclear	Children Plaque psoriasis (53%)	Male: 47% Female: 53%	4.3% (6/137)		High risk of bias	Yes

*Note:* Prevalence of eczema in psoriasis populations.

Abbreviations: CI, confidence interval; IgE, immunoglobulin E; ISAAC, International Study of Asthma and Allergies in Childhood; HLA, human leucocyte antigen; OR, odds ratio; RAST, radioallergosorbent.

^a^ Study duplicated in Tables 1 and 2 because the study contributes data from both a psoriasis and an eczema population.

**TABLE 2:** Study characteristics of included studies

Study, year, country	Study type and setting	Aim	Diagnosis	Sample size (*n* = eczema population)	Timing of outcome	Population	Male and female (%)	Prevalence of psoriasis (%)	Other key findings	Risk of bias	Blinded assessment of outcome diagnosis
Abramovits et al.^15^ ^,^ ^a^ 2005, USA	Cross‐sectional hospital‐based Dermatology department	To profile clinical patients with eczematous dermatitis on the palms and ventral areas of the digits	Psoriasis: Self‐reported Eczema: healthcare professional (physician)	50	One time point Any time point	Adults and children Hand eczema	Male: 52% Female: 48%	One time point 6% (3/50) Any time point 12% (6/50)		High risk of bias	No
Barry et al.^18^ ^,^ ^a^ 2019, USA	Cross‐sectional hospital‐based Dermatology department	To characterise the population of patients diagnosed with both atopic dermatitis and psoriasis	Psoriasis and eczema: health professional (not specified) and histology (33%)	912	One time point Any time point	Adults and children	Male and female: % unclear	At one time point 0.3% (3/912) At any time point 3%	Concomitant 1.3% (30/2303)	High risk of bias	Yes
Beer et al.^12^ ^,^ ^a^ 1992, UK	Cross‐sectional hospital‐based Dermatology department	A prospective study to record the concurrent or consecutive coincidence of psoriasis and atopic dermatitis, and any shared clinical features	Eczema and psoriasis: dermatologist (criteria for diagnosis specified)	224	One time point Any time point	Adults and children	Male: 76% Female: 24%	At one time point 12.6% (34/269) At any time point 16.7% (45/269)		Low risk of bias	No
Drewitz et al.^22^ Germany, 2019	Cohort population‐based Regional area of Germany Conference abstract	To investigate the prevalence of comorbidities in atopic and hand eczema	Psoriasis and eczema: self‐reported	37	Any time point	Elderly adults (>70 years)	Male: 41% Female: 59%	13.5%	Prevalence in controls: 5.3%	High risk of bias	Yes
Henseler & Christophers,^10^ ^,^ ^a^ 1995, Germany	Cross‐sectional hospital‐based Dermatology department	To determine the frequency of concurrent diseases	Psoriasis and eczema: dermatologist	1701	One time point	Age not specified Hospitalised	Male: 50% Female: 50%	0.29% (5/1701)		Low risk of bias	Yes
Kim et al.^27^ ^,^ ^a^ 2010, South Korea	Cross‐sectional population‐based Health centre attendance for an annual health check	To determine the prevalence of atopic dermatitis in Korean adults and also the coincidence of psoriasis	Psoriasis: dermatologist and self‐ reported Eczema: dermatologist and self‐ reported, both based on Hanifin and Rajka criteria	52	Any time point	Adults Korean ethnicity	Male and female: % unclear	19.2% (10/52)	Prevalence coexistent disease in the whole population 0.5% (10/2032) Odds ratio of psoriasis in eczema 4.8	Low risk of bias	No
Krishna et al.^28^ 2019, UK	Cohort population‐based THIN database—a database of primary care records	To investigate whether allergic disease increases the incidence of autoimmune disease	Psoriasis and eczema: electronic read codes (health professional not specified)	1 393 570	Any time point	Adults and children	Male: 46% Female: 54%	2.89% (40, 319/1 393 570)	Adjusted incidence rate ratio of psoriasis in eczema: 2.41 (95% CI 2.36, 2.46)	Low risk of bias	Yes
Lambert & Dalac,^29^ ^,^ ^a^ 1992, France & Belgium	Cross‐sectional hospital‐based Dermatology department	To look at the association between psoriasis and eczema	Psoriasis: dermatologist Eczema: dermatologist using diagnostic criteria	3494	Unclear	Age not specified	Male and female: % unclear	2.7% (95/3494)		High risk of bias	No
Naldi et al.^30^ ^,^ ^a^ 2009, Italy	Cross‐sectional community‐based Secondary schools	To estimate the lifetime risk of atopic dermatitis in children and to quantify the association between selected diseases	Psoriasis and eczema: health professional (from the medical record)	202	Any time	Children (12–17 years) Attending school Caucasian	Male: 55% Female: 45%	7.9% (16/202)		Low risk of bias	Yes
Simpson et al.^34^ 2002, UK	Cross‐sectional population‐based CMR database—a database of primary care records	To investigate if TH1 and TH2 disease are associated or mutually exclusive	Psoriasis and eczema diagnosis: electronic read codes (healthcare professional)	7035	One time point	Adults and children	Male and female: % unclear		Relative risk of psoriasis in eczema 2.88 (95% CI: 2.38, 3.45)	Low risk of bias	Yes
Stepanova et al.^35^ ^,^ ^a^ 2004, Germany	Cross‐sectional hospital‐based Dermatology department	To report the occurrence of ‘mixed forms’ of eczema and psoriasis, and verify the immunological relationship, total IgE value and distribution of the HLA characteristics	Psoriasis: diagnostic criteria (not specified who made the diagnosis)^b^ Eczema: Hanifin and Rajka (not specified who made the diagnosis)^b^ ‘Mixed form’ defined as ‘atopic dermatitis criteria’ as well as the ‘psoriasis criteria’	237	One time point	Adults Hospitalised	Male and female: % unclear	6.3% (15/237)	42.5% (34/80) of ‘mixed form’ have elevated total IgE >120 kU/L ‘Mixed form’ in females demonstrated homozygosity of the region between HLA‐B and DRB1: DRB4 (DR53)	High risk of bias	No
Williams et al.^39^ 1994, UK	Cohort population‐based National child development survey—birth cohort	To determine whether psoriasis and eczema could exist simultaneously and consecutively in the same individuals	Psoriasis and eczema: healthcare professional (not specified) assessing for visible disease	354	One time point Any time point	Children (11–16 years)	Male and female: % unclear	One time point age 11%–1.3% (3/223) age 16%–1.3% (3/234) Any time point 1.4% (5/354)	Relative risk at any time point 1.41 (95% CI: 0.58, 3.46)	Low risk of bias	Yes
Zander et al.^40^ 2020, Germany	Cross‐sectional population‐based Occupational skin cancer screening	To determine robust data on the prevalence of dermatological comorbidity in people with atopic dermatitis	Psoriasis: dermatologist Eczema: dermatologist using Hanifin and Rajka criteria and self‐reported	1724	One time point Any time point	Adults	Male: 59% Female: 41%	At one time point 1.22% (95% CI: 0.75, 1.86) At any time point 3.03% (95% CI: 2.60, 3.50)	Adjusted odds ratio 0.61 (95% CI: 0.39, 0.94), adjusted for age, gender, and skin type	Low risk of bias	Yes

*Note:* Prevalence of psoriasis in eczema populations.

Abbreviations: CI, confidence interval; IgE, immunoglobulin E; HLA, human leucocyte antigen.

^a^ Study duplicated in Tables 1 and 2 because the study contributes data from both a psoriasis and an eczema population.

^b^ method of diagnosis only specified for exposure.

The total number of individuals in the psoriatic exposure population were 20 523 (range: 52–3837, median: 306) and in the eczema exposure population were 1 405 911 (range: 37–1 393 570, median: 354).

### 4.1 Primary objective

#### 4.1.1 Prevalence of eczema in psoriasis studies

Eight studies reported the prevalence of eczema in a psoriasis population, where the diseases occurred at the same time point. The main results for each study are presented in Table 1. The prevalence ranged from 0.17%^10^ to 20%.^15^ The pooled prevalence estimate of eczema in those with psoriasis at the same time point was 2% (95% CI: 1, 3) (Figure 2). There was evidence of high statistical heterogeneity between the eight studies (*I* ^2 ^= 93.4%). Forest plots exploring heterogeneity are presented in Figure 2 and Supporting Information 4. Geographical area and method of diagnosis may help explain the observed heterogeneity for the pooled prevalence of eczema in those with psoriasis; a higher pooled prevalence was observed in Europe (2%, 95% CI: 1, 4%) and when a diagnosis was made by a dermatologist/using diagnostic criteria (3%, 95% CI: 1, 5). However, these results were heavily influenced by Abramovits et al.^15^ a small cross‐sectional study of 100 patients with psoriasis.

FIGURE 2

**Figure:**
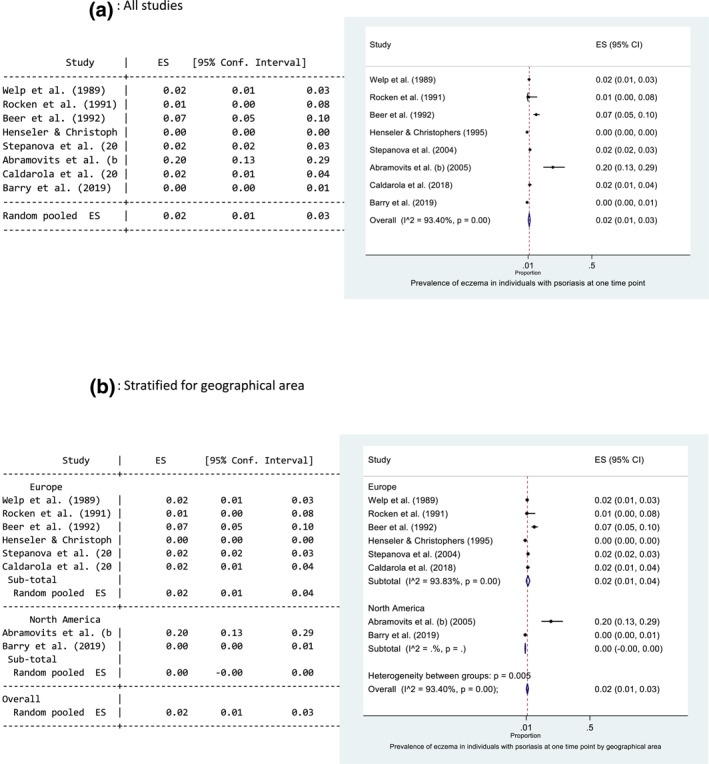

**Figure:**
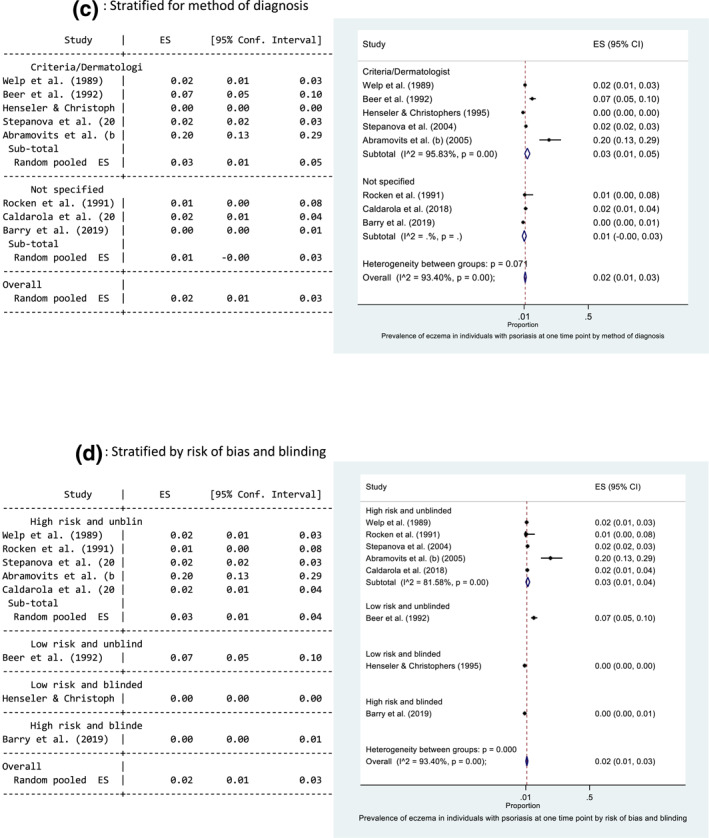
Forest plots of the prevalence of eczema in individuals with psoriasis at one time point. All studies (a), and stratified for geographical area (b), method of diagnosis (c), low risk of bias and blinding (d). Proportion of 0.1 represents a prevalence of 10%

Only two studies were rated as low risk of bias, and only one of these provided data where the outcome diagnosis (eczema) was made blinded to the research question.^10^ Henseler et al.^10^ used mostly prospectively collected data from a large secondary care database of 42 461 patients at one dermatology department. Diagnoses were made by a minimum of two experienced dermatologists. The study aimed to investigate the coexistence of psoriasis and more than 50 other skin diseases, and the diagnoses were made blinded to the study question. Beer et al.^12^ examined 983 consecutive patients attending the dermatology department. The diagnoses were made by one consultant dermatologist who was leading the study investigating the coexistence of eczema and psoriasis. There was a large difference between the prevalence values of these two studies (0.17% vs. 7.2%), indicating that when the outcome diagnosis was made by a clinician who was looking for coexistent disease the prevalence values are higher (Figure 2).

#### 4.1.2 Prevalence of psoriasis in eczema populations

Seven studies reported the prevalence of psoriasis in an eczema population, where the diseases occurred at the same time point. The main results from each study are presented in Table 2. The prevalence ranged from 0.3%^18^ to 12.6%.^12^ The pooled prevalence estimate of psoriasis in those with eczema at the same time point was 2% (95% CI: 1, 3) (Figure 3). There was evidence of high statistical heterogeneity between the seven studies (*I* ^2 ^= 90.53%). Forest plots exploring heterogeneity are presented in Figure 3 and Supporting Information 4. There is evidence that setting may help explain the observed heterogeneity. Heterogeneity remained high between the hospital‐based studies (*I* ^2 ^= 92.60%) but was greatly reduced between the population‐based studies (Figure 3).

FIGURE 3Forest plots of the prevalence of psoriasis in individuals with eczema at one time point. All studies (a), and stratified for setting (b) and low risk of bias and blinding (c). Proportion of 0.1 represents a prevalence of 10%

**Figure:**
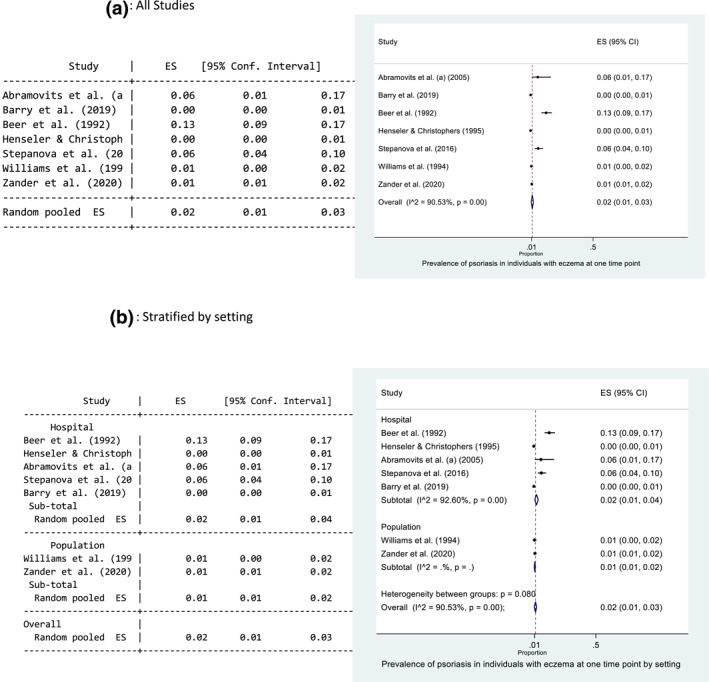

**Figure:**
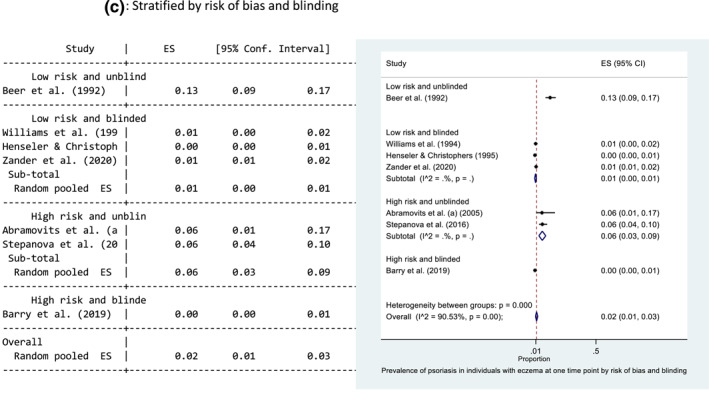

Five studies were rated as low risk of bias, and four provided data where the outcome diagnosis (psoriasis) was made blinded to the research question.^10^ ^,^ ^34^ ^,^ ^39^ ^,^ ^40^ These four studies include Beer et al. and Henseler et al.^10^ ^,^ ^12^ Williams et al.^39^ conducted a cohort study of 9263 children using data from the National Child Development Survey 1958. Diagnoses were made by medical officers, blinded to the research question being posed many years later. Zander et al.^40^ included data from 118 939 individuals included in standardised occupational skin examinations by trained dermatologists. These diagnoses were made blinded to the research question of the study. Simpson et al.^34^ included 7035 with eczema in a cross‐sectional study using electronic primary care records and found the relative risk of psoriasis in the eczema population to be 2.88 (95% CI: 2.38, 3.45). Definitive doctor diagnoses were recorded as codes before the conduct of this study. Similar to the findings in psoriasis patients, the pooled prevalence of psoriasis in those with eczema was lower in studies where the diagnosis was made blinded to the research question (1% vs. 13%) (Figure 3).

### 4.2 Secondary objective

**FIGURE 4:**
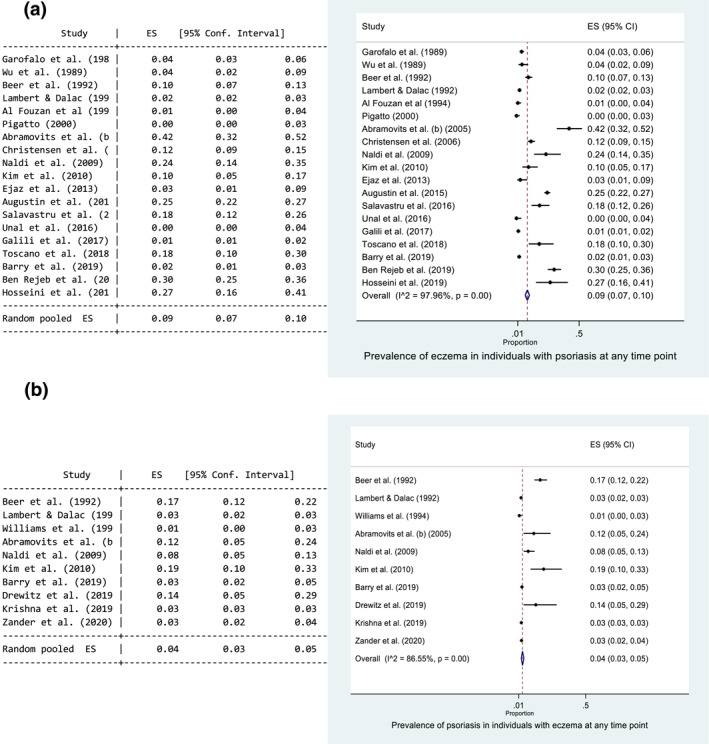
a) Forest plot of the prevalence of eczema in individuals with psoriasis at any time point. (b) Forest plot of the prevalence of psoriasis in individuals with eczema at any time point. Proportion of 0.1 represents a prevalence of 10%

The secondary objective investigated coexistence at any time point. Seventeen studies reported the prevalence of eczema in a psoriasis population at any time point. The main results for each study are provided in Table 1. The pooled prevalence of eczema in those with psoriasis at any time point was 9% (95% CI: 7, 10) (Figure 4). Ten studies reported the prevalence of psoriasis in an eczema population. The main results for each study are presented in Table 2.The pooled prevalence of psoriasis in those with eczema at any time point was 4% (95% CI: 3, 5) (Figure 4).

One study reported the odds and two studies reported the relative risk of eczema coexisting in a psoriasis population compared to the general population. Galili et al.^24^ found the adjusted OR of eczema in those with psoriasis to be 1.75 (95%CI 1.29, 2.37).^24^ Landgren et al. and Naldi et al. reported the relative risk of eczema in those with psoriasis to be 0.4 (95%CI 0.30, 0.55)^11^ and 5.5 (95%CI 3.0, 10.1)^30^ respectively. Both studies were rated as low risk of bias and the outcome diagnosis was made blinded, but included different populations, military conscripts versus children.

Two studies reported the odds and one study reported the relative risk of psoriasis coexisting in an eczema population compared to the general population. Zander et al. reported an adjusted OR: 0.61 (95% CI: 0.39, 0.94) and Kim et al.^27^ an OR 4.8 (*n* = 2042). The relative risk was reported as 1.41 (95% CI: 0.58, 3.46) by Williams et al.^39^ The two studies reporting a smaller association, Zander et al. and Williams et al. were both rated low risk of bias and the outcome diagnosis was made blinded.

### 4.3 Tertiary objective

Stepanova et al.^35^ reported immunological and genetic findings on the study population. Of individuals with a ‘mixed form’ of eczema and psoriasis, 42.5% (34/80) had an elevated total immunoglobulin E (IgE) of over 120 kU/L and females demonstrated homozygosity of the region between human leucocyte antigen B (HLA‐B) and DRB1: DRB4 (DR53). Welp et al.^38^ also reported laboratory findings. Of those with coexistent disease, 89% (16/18) of the patients showed a total IgE level of over 120 IU/ml and there was a significant increase in HLA‐Cw6 in 50% (9/18) patients, while HLA‐B7 and HLA‐B17 were only found in 11% (2/18) patients.

## 5 DISCUSSION

### 5.1 Main findings

This review provides some evidence for the coexistence of atopic eczema and psoriasis, and no convincing evidence for the complete mutual exclusivity of both diseases. The pooled prevalence results for both populations were 2%, indicating coexistence of disease may occur at a level equal or lower than expected. There were seven studies rated low risk of bias where the outcome diagnosis was made blinded to the research question, but the findings from these studies are not consistent. Prevalence at one time point may be lower than expected (0.17% for eczema in those with psoriasis and 1% for psoriasis in those with eczema),^10^ ^,^ ^39^ ^,^ ^40^ but the relative risk for psoriasis occurring in those with atopic eczema was estimated to be 2.88.^34^ At any time point, results for the relative occurrence of atopic eczema in those with psoriasis and psoriasis in those with atopic eczema were conflicting, RR = 0.4 and RR = 5.5^11^ ^,^ ^30^ and 0.61 and 1.41,^39^ ^,^ ^40^ respectively.

### 5.2 Existing literature

There is both immunological and genetic reasoning for why coexistence could be biologically plausible. Historically, it was proposed that eczema and psoriasis are driven by opposing T‐cell activation pathways.^42^ However, the activation of Th2 and Th1 cells is likely to be antigen specific and not related to the immune system as a whole.^34^ Studies have indicated that activation of specific T‐cell pathways and cytokines may not be exclusive to each disease. Th17 and Th22 are typically associated with psoriasis, but have been found to have a comparatively smaller role in chronic eczema.^43^ In both diseases, they stimulate a keratinocyte response and epidermal thickening. This immunological crossover may be more common in children and certain disease phenotypes such as Asian atopic dermatitis.^44^

Genetically, there has been shown to be an overlap in the susceptibility foci, genetic pathways and genomic regulatory sites in allergic and autoimmune disease.^45^ For example, chromosomes 1q21, 17q25 and 20p loci identified in the UK genome screen for eczema coincide with genes which are associated with psoriasis.^46^ The area of the most overlap on chromosome 1q21 is the region which overlies the epidermal differentiation complex. Additionally, a study in this review, Stepanova et al.^35^ found evidence of homozygosity in the region between HLA‐B and HLA‐DRB1: DRB4 (DR53), this area determines HLA class III characteristics such as tumour necrosis factor‐*α* and *β* ^22^ and certain complement factors which are involved in both diseases.^35^

### 5.3 Strengths and limitations

One of the strengths of this review is the large total sample size of over 1.4 million individuals with eczema and psoriasis. The review includes studies published in English, German and French, but most studies included populations from North America and Western Europe, potentially limiting the generalisability of the findings to multiple ethnic groups. In the critical appraisal of each study, the review team agreed that a study could be defined as low risk of bias if it scored 70% or above. This threshold is arbitrary and affects which studies were included in the sensitivity analyses.

The conclusions of the review are limited by studies identified. There were important differences between studies, for example data source and methodology, and this led to high heterogeneity in the meta‐analyses. The observed heterogeneity for the pooled prevalence of eczema in those with psoriasis were heavily influenced by Abramovits et al.,^15^ a small cross‐sectional study of 100 patients with psoriasis. Despite looking at subgroups of studies, it was difficult to provide useful pooled values, often with sparse data or still notably different results for studies within the same strata.

Included studies used both existing data (retrospective) from departmental and national databases, and purposefully collected prospective data mostly from hospital dermatology departments. Both types of data have advantages and disadvantages. The recording of diagnoses in retrospective sources may have considered the diagnoses to be mutually exclusive, whereas unless there was blinding of the assessment, prospective collection could have been biased by the known aims of the study: leading to under or over reporting of coexistence. We have shown that the prevalence of coexistence was reported lower in studies where the outcome diagnosis was made blinded to the research question.

There were many studies with a high risk of bias (*n* = 21). A key critical appraisal point, and vital for the interpretation of the study, was specifying how the diagnoses was made in a valid and reliable way. We did not make assumptions if this information was not clear. Studies which used validated diagnostic criteria, or a dermatologist's diagnosis detailing criteria if no others available, provided higher quality evidence. This was more likely to occur in studies based in hospital dermatology departments. The validity of diagnoses in national databases were challenging to critically appraise and we relied on authors specifying that the diagnostic codes had been validated.

Included studies also mostly reported prevalence data and did not compare to a control group. The absence of a baseline comparator limits the interpretation of the data because population prevalence of both eczema and psoriasis differs depending on age, geographical area and setting.

The division between eczema and psoriasis may not be absolute and there is a growing body of research into ‘mixed disease’, challenging how eczema and psoriasis are categorised into distinct entities. It may also be argued that the data to support coexistence represents misdiagnosis, especially when existing data sources are used, however this does not explain the coexistence results from prospective studies assessing for simultaneous disease.

### 5.4 Implications

Clinicians should be aware that there is evidence for the coexistence of atopic eczema and psoriasis and be receptive to the emergence of new and evolving skin changes; potentially re‐evaluating and adding a second diagnosis of a common disease. If coexistence is identified, and the skin involvement is not controlled, this may alter the therapeutic approach. At a simple level this may include using vitamin D analogues or tar products if there is evidence of psoriasis in a patient previously diagnosed with eczema. Identifying coexistence may help direct the choice of therapies to one's that have efficacy in both diseases, for example the use of light therapy or methotrexate, or newer Janus kinase inhibitors. When using biologic therapies, it may also be useful to consider the effect these might have on differing immunological pathways for patients with coexistent diseases.

## CONFLICT OF INTERESTS

The authors declare that there are no conflict of interests.

## Supporting information

Supplementary Material

Supplementary Material

Supplementary Material

Supplementary Material

## Data Availability

The data that support the findings of this study are available from the corresponding author upon reasonable request.
