# Supplementary material for: Can atopic eczema and psoriasis coexist? A systematic review and meta‐analysis
Source: Skin Health Dis. 2021 May 5;1(2):e29. doi: 10.1002/ski2.29 (PMC9060081; doi:10.1002/ski2.29)
Supplement: Supplementary file 1 — Supplementary Material [file SKI2-1-e29-s003.docx]

Supplementary 1: Seach strategy

**Ovid MEDLINE**

1 psoriasis.mp.

2 PSORIASIS/

3 1 or 2

4 eczema.mp.

5 atopic dermatitis.mp.

6 neurodermatitis.mp.

7 ECZEMA/

8 exp DERMATITIS, ATOPIC/

9 4 or 5 or 6 or 7 or 8

10 comorbidity.mp.

11 comorbidities.mp.

12 comorbid.mp.

13 co-morbidity.mp.

14 co-morbidities.mp.

15 co-morbid.mp.

16 coexistence.mp.

17 co-existence.mp.

18 coexisting.mp.

19 co-existing.mp.

20 coexist.mp.

21 co-exist.mp.

22 concurrent.mp.

23 concurrence.mp.

24 concurring.mp.

25 concomitant.mp.

26 concomitance.mp.

27 overlap.mp.

28 overlapping.mp.

29 accompanying.mp.

30 association.mp.

31 associated.mp.

32 exp Comorbidity/

33 multimorbidity.mp.

34 multimorbidities.mp.

35 multi-morbidity.mp.

36 multi-morbidities.mp.

37 risk.mp.

38 incidence.mp.

39 prevalence.mp.

40 or/10-39

41 3 and 9 and 40

**Ovid Embase**

1 psoriasis.mp.

2 exp psoriasis/

3 1 or 2

4 eczema.mp.

5 atopic dermatitis.mp.

6 neurodermatitis.mp.

7 exp eczema/

8 exp atopic dermatitis/

9 4 or 5 or 6 or 7 or 8

10 comorbidity.mp.

11 comorbidities.mp.

12 comorbid.mp.

13 co-morbidity.mp.

14 co-morbidities.mp.

15 co-morbid.mp.

16 coexistence.mp.

17 co-existence.mp.

18 coexisting.mp.

19 co-existing.mp.

20 coexist.mp.

21 co-exist.mp.

22 concurrent.mp.

23 concurrence.mp.

24 concurring.mp.

25 concomitant.mp.

26 concomitance.mp.

27 overlap.mp.

28 overlapping.mp.

29 accompanying.mp.

30 association.mp.

31 associated.mp.

32 Comorbidity/

33 multimorbidity.mp.

34 multimorbidities.mp.

35 multi-morbidity.mp.

36 multi-morbidities.mp.

37 risk.mp.

38 incidence.mp.

39 prevalence.mp.

40 or/10-39

41 3 and 9 and 40
