# Supplementary material for: Can atopic eczema and psoriasis coexist? A systematic review and meta‐analysis
Source: Skin Health Dis. 2021 May 5;1(2):e29. doi: 10.1002/ski2.29 (PMC9060081; doi:10.1002/ski2.29)
Supplement: Supplementary file 2 — Supplementary Material [file SKI2-1-e29-s001.docx]

Supplementary 3: Excluded studies

| Study details | Reason excluded |
| --- | --- |
| Response to Letter to the editor: 'Psoriasis dermatitis: an overlap condition of psoriasis and atopic dermatitis in children'. Docampo *et al.* 2019 | Not an observational study |
| Clinical characteristics, symptoms and burden of psoriasis and atopic dermatitis in adults. Egeberg *et al.* 2020 (epub 2019). | No data on co-existence |
| Eyelid dermatitis: experience in 203 cases. Guin 2002. | No data on co-existence |
| A comparative study of childhood psoriasis and atopic dermatitis and greater understanding of the overlapping condition, psoriasis-dermatitis. Kapila *et al.* 2012. | No data on co-existence |
| Epidemiology and dermatological comorbidity of seborrhoeic dermatitis: population-based study in 161 269 employees. Zander *et al.* 2019. | Overlapping study (duplicate) |
| A genome-wide association study of atopic dermatitis identifies loci with overlapping effects on asthma and psoriasis. Weidinger *et al.* 2004 | No data on co-existence |
| Contrasting disease patterns in psoriasis and atopic dermatitis. Christopher and Henseler, 1987. | Overlapping study (duplicate) |
| Co-existence of psoriasis and atopic dermatitis in same patients and families. Konathan and Kumar, 2014. | Not an observational study (<10 patients) |
| Psoriasis: epidemiology and clinical spectrum. Christophers 2001. | Not an observational study |
| Comorbidities of atopic dermatitis. Cribier, 2019. | Not an observational study |
| Psoriasis and atopic dermatitis: Coincidence or association? Sinsi et al 1990 | Unable to access full-text |
| Questionable coexistence of TH1- and TH2- related diseases. Benn *et al.* 2002. | Not an observational study |
| Survey of comorbidities in patients with psoriasis in Slovakia. Chromej *et al.* 2011. | No data on co-existence. |
| Concomitance of psoriasis and atopic dermatitis--a relative phenomenon. Dhar *et al.* 1993. | Not an observational study. |
| Comorbidities in autoimmune skin diseases. Linder *et al.* 2018. | Not an observational study |
| Prevalence and comorbidities in adults with psoriasis compared to atopic eczema. Radtke *et al.* 2017. | No data on co-existence. |
| Co-occurrence and comorbidities in patients with immune-mediated inflammatory disorders: An exploration using US healthcare claims data, 2001-2002. Robinson *et al.* 2006. | No data on co-existence |
| Dermatological screening of a middle-aged and elderly population: the Rotterdam Study. Sanders *et al.* 2017 | No data on co-existence |
| Comparative analysis of comorbidity in children with psoriasis and atopic eczema. Schafer *et al.* 2014. | Insufficient or unclear data |
| Psoriasis comorbidities:5-year retrospective study. Solovan *et al.* 2011. | Insufficient or unclear data |
