# Supplementary material for: Can atopic eczema and psoriasis coexist? A systematic review and meta‐analysis
Source: Skin Health Dis. 2021 May 5;1(2):e29. doi: 10.1002/ski2.29 (PMC9060081; doi:10.1002/ski2.29)
Supplement: Supplementary file 3 — Supplementary Material [file SKI2-1-e29-s002.pdf]

Supplementary

Forest plot of the prevalence of eczema in individuals with psoriasis at one time point stratified for age (a) and risk of bias (b).
All studies were from a hospital setting.

(a) Age

Study	ES	[95% Conf.	Interval]
Adults			
Welp et al. (1989)	0.02	0.01	0.03
Beer et al. (1992)	0.07	0.05	0.10
Henseler & Christoph	0.00	0.00	0.00
Stepanova et al. (20	0.02	0.02	0.03
Abramovits et al. (b	0.20	0.13	0.29
Caldarola et al. (20	0.02	0.01	0.04
Barry et al. (2019)	0.00	0.00	0.01
Sub-total			
Random pooled ES	0.02	0.01	0.03
Unclear			
Rocken et al. (1991)	0.01	0.00	0.08
Overall			
Random pooled ES	0.02	0.01	0.03

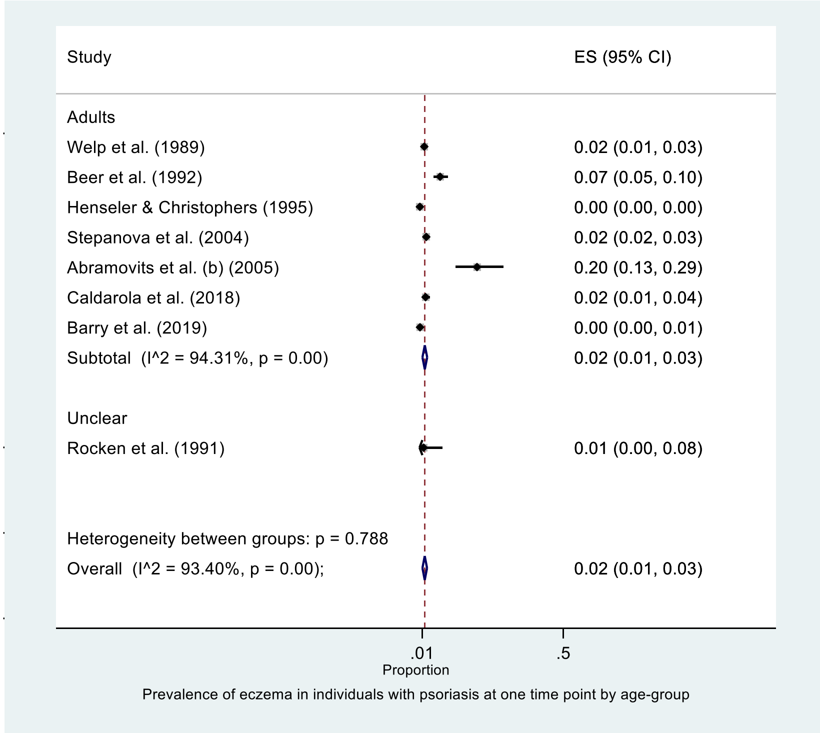

(b) Risk of bias

Study	ES	[95% Conf.	Interval]
High			
Welp et al. (1989)	0.02	0.01	0.03
Rocken et al. (1991)	0.01	0.00	0.08
Stepanova et al. (20	0.02	0.02	0.03
Abramovits et al. (b	0.20	0.13	0.29
Caldarola et al. (20	0.02	0.01	0.04
Barry et al. (2019)	0.00	0.00	0.01
Sub-total			
Random pooled ES	0.02	0.01	0.04
Low			
Beer et al. (1992)	0.07	0.05	0.10
Henseler & Christoph	0.00	0.00	0.00
Sub-total			
Random pooled ES	0.00	0.00	0.00
Overall			
Random pooled ES	0.02	0.01	0.03

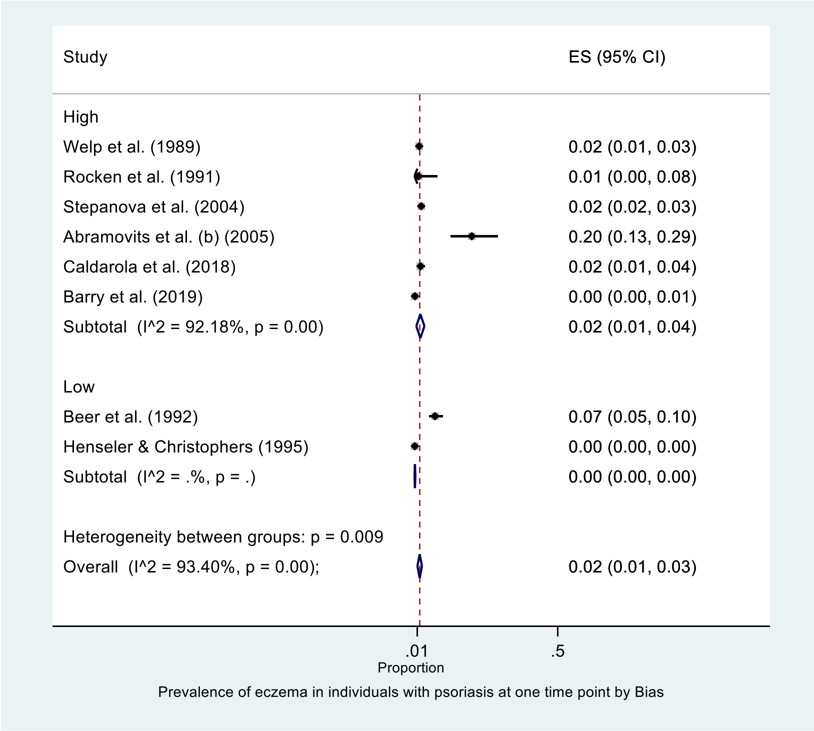

Forest plot of the prevalence of psoriasis in individuals with eczema at one time point stratified for age (a), risk of bias (b), geographical area (c), method of diagnosis (d).

(a) Age

Study	ES	[95% Conf.	Interval]
Adults			
Abramovits et al. (a	0.06	0.01	0.17
Barry et al. (2019)	0.00	0.00	0.01
Beer et al. (1992)	0.13	0.09	0.17
Stepanova et al. (20	0.06	0.04	0.10
Zander et al. (2020)	0.01	0.01	0.02
Sub-total			
Random pooled ES	0.04	0.02	0.05
Unclear			
Henseler & Christoph	0.00	0.00	0.01
Children			
Williams et al. (199	0.01	0.00	0.02
Overall			
Random pooled ES	0.02	0.01	0.03

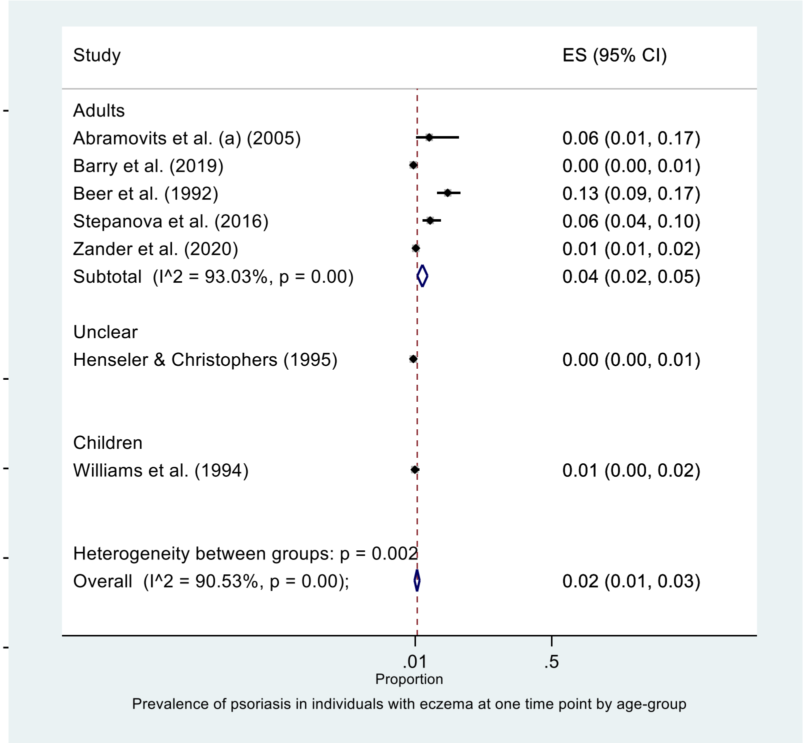

(b) Risk of bias

Study	ES	[95% Conf.	Interval]
High			
Abramovits et al. (a	0.06	0.01	0.17
Barry et al. (2019)	0.00	0.00	0.01
Stepanova et al. (20	0.06	0.04	0.10
Sub-total			
Random pooled ES	0.04	-0.01	0.09
Low			
Beer et al. (1992)	0.13	0.09	0.17
Henseler & Christoph	0.00	0.00	0.01
Williams et al. (199	0.01	0.00	0.02
Zander et al. (2020)	0.01	0.01	0.02
Sub-total			
Random pooled ES	0.02	0.00	0.03
Overall			
Random pooled ES	0.02	0.01	0.03

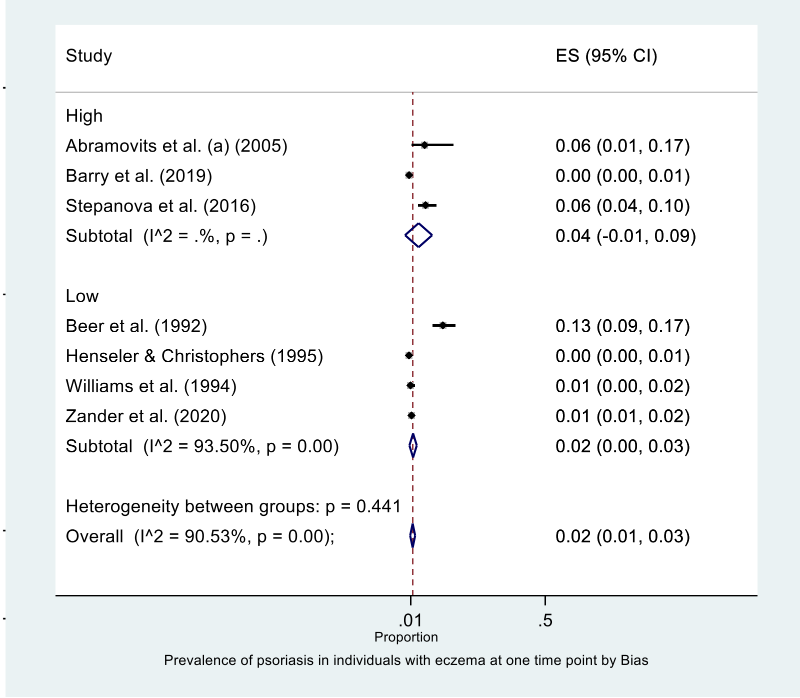

(c) Geographical area

Study	ES	[95% Conf.	Interval]
**North America**			
Abramovits et al. (a	0.06	0.01	0.17
Barry et al. (2019)	0.00	0.00	0.01
Sub-total			
Random pooled ES	0.00	-0.00	0.01
**Europe**			
Beer et al. (1992)	0.13	0.09	0.17
Henseler & Christoph	0.00	0.00	0.01
Stepanova et al. (20	0.06	0.04	0.10
Williams et al. (199	0.01	0.00	0.02
Zander et al. (2020)	0.01	0.01	0.02
Sub-total			
Random pooled ES	0.02	0.01	0.04
Overall			
Random pooled ES	0.02	0.01	0.03

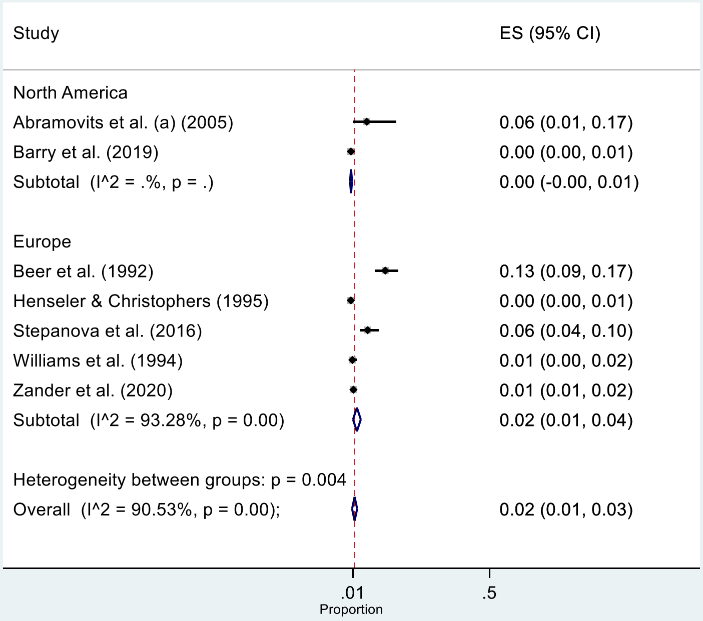

Prevalence of psoriasis in individuals with eczema at one time point by geographical area

(d) Method of diagnosis

Study	ES	[95% Conf.	Interval]
**Criteria/Dermatologi**			
Beer et al. (1992)	0.13	0.09	0.17
Henseler & Christoph	0.00	0.00	0.01
Stepanova et al. (20	0.06	0.04	0.10
Zander et al. (2020)	0.01	0.01	0.02
Sub-total			
Random pooled ES	0.03	0.01	0.05
**Healthcare**			
Williams et al. (199	0.01	0.00	0.02
Abramovits et al. (a	0.06	0.01	0.17
Sub-total			
Random pooled ES	0.01	0.00	0.02
**Not specified**			
Barry et al. (2019)	0.00	0.00	0.01
Overall			
Random pooled ES	0.02	0.01	0.03

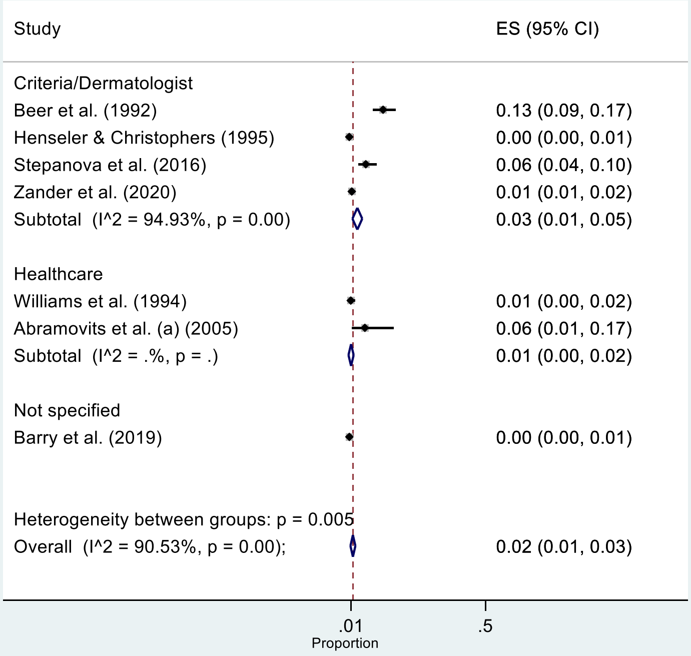

Prevalence of psoriasis in individuals with eczema at one time point by method of diagnosis
