# Supplementary material for: Can atopic eczema and psoriasis coexist? A systematic review and meta‐analysis
Source: Skin Health Dis. 2021 May 5;1(2):e29. doi: 10.1002/ski2.29 (PMC9060081; doi:10.1002/ski2.29)
Supplement: Supplementary file 4 — Supplementary Material [file SKI2-1-e29-s004.docx]

Supplementary 2: Data extraction Table

| **Title** |  |
| --- | --- |
| **Lead author** |  |
| **Year of publication** |  |
| **Country of the study** |  |
| **Study period** |  |
| **Study aim (either specified in or inferred from the paper)** |  |
| **Study type**  **(circle as appropriate)** | Case series, cross sectional, case-control, cohort (prospective or retrospective) |
| **Study population**  **(circle as appropriate)** | Age: Adults, children (<18 years), adults and children  Exposure: Eczema, Psoriasis  Any further details:  Gender:  Ethnicity:  Socioeconomic status:  Environmental exposure:  Psoriasis subtype:  Eczema subtype: |
| **Outcome**  **(circle as appropriate)** | Eczema, Psoriasis, Psorisi-dermatitis |
| **Diagnosis**  **(circle as appropriate)** | Psoriasis diagnosis – clinical by a dermatologist, clinical by another health professional, self-diagnosis, not specified  Eczema diagnosis – diagnostic criteria, clinical by a dermatologist, clinical by another health professional, self-diagnosis, not specified |
| **Sample size** | Cases:  Controls (if applicable): |
| **Key findings**  **(prevalence, incidence, risk – OR/relative risk)**  **Psorisi-dermatitis** |  |
| **Strengths/limitations**  **Authors and reviewer** |  |
| **Evidence of coexistence between eczema and psoriasis (genetics and immunology)** |  |
| **Timing of outcome** | Any time point, one time point, unclear |
